# *In vitro*-generated interspecific recombinants between bovine herpesviruses 1 and 5 show attenuated replication characteristics and establish latency in the natural host

**DOI:** 10.1186/1746-6148-7-19

**Published:** 2011-05-18

**Authors:** Maria P Del Medico Zajac, Sonia A Romera, María F Ladelfa, Fiorella Kotsias, Fernando Delgado, Julien Thiry, François Meurens, Günther Keil, Etienne Thiry, Benoît Muylkens

**Affiliations:** 1 Virology Institute, Veterinary and Agricultural Science Research Center, National Institute of Agricultural Technology, Hurlingham, Buenos Aires, Argentina; 2 Virology and Viral Diseases, Department of Infectious and Parasitic Diseases, Faculty of Veterinary Medicine, University of Liege, Liege, Belgium; 3 Institut National de la Recherche Agronomique (INRA), Infectiologie Animale et Santé Publique, Nouzilly (Tours), France; 4 Friedrich-Loeffler-Institut, Federal Research Centre for Animal Health, Riems, Germany; 5 Pathobiology Institute, Veterinary and Agricultural Science Research Center, National Institute of Agricultural Technology, Hurlingham, Buenos Aires, Argentina; 6 Consejo Nacional de Investigaciones Científicas y Tecnológicas (CONICET), Ciudad de Autónoma de Buenos Aires, Argentina

## Abstract

**Background:**

Interspecific recombinant viruses R1ΔgC and R2ΔgI were isolated after *in vitro *co-infection with BoHV-1 and BoHV-5, two closely related alphaherpesviruses that infect cattle. The genetic characterization of R1ΔgC and R2ΔgI showed that they are composed of different sections of the parental genomes. The aim of this study was the characterization of the *in vivo *behavior of these recombinants in the natural host.

**Results:**

Four groups of four 3-month-old calves of both genders were intranasally inoculated with either the recombinant or parental viruses. A control group of two animals was also included. Viral excretion and clinical signs were monitored after infection. Histopathological examination of the central nervous system (CNS) was performed and the establishment of latency in trigeminal ganglia was analyzed by PCR. The humoral response was also evaluated using ELISA tests.

Three out of four animals from the BoHV-5 infected group excreted virus for 4-10 days. Two calves shed R1ΔgC virus for one day. In R2ΔgI and BoHV-1.2ΔgCΔgI groups, infectious virus was isolated only after two or three blind passages. None of the infected animals developed neurological signs, although those infected with BoHV-5 showed histopathological evidence of viral infection. Latent viral DNA was detected in at least one calf from each infected group. Serum and/or mucosal antibodies were detected in all groups.

**Conclusion:**

Both BoHV-1/-5 recombinants and the BoHV-1 parental strain are attenuated in calves, although they are able to replicate in animals at low rates and to establish latent infections.

## Background

Bovine herpesviruses 1 (BoHV-1) and 5 (BoHV-5) are two closely related alphaherpesviruses infecting cattle. BoHV-1 respiratory infection is usually associated with infectious bovine rhinotracheitis, abortion, and systemic infection in neonates [1]. BoHV-5 infection induces respiratory and nervous diseases, i.e. meningoencephalitis [2]. Both viruses are neurotropic and establish a life-long latent infection in neurons of sensory ganglia [2,3].

BoHV-1 infection is present in all continents, although there are differences in prevalence and incidence [4]. In the USA, Canada, Australia and New Zealand the seroprevalence of BoHV-1 infection is variable but may be very high. BoHV-1 is also present in European countries, where a variety of control programs are carried out. Concerning South American countries, BoHV-1 infection is endemic and the seroprevalence is high [5]. On the other hand, BoHV-5 outbreaks are sporadic and appear to be very restricted in their geographical distribution, being mostly detected in the Southern hemisphere [2]. In Argentina and Brazil, BoHV-1 and BoHV-5 co-circulate and vaccination against BoHV-1 is not obligatory. In addition, cattle co-infection has been recently shown to exist [6]. In this context, *in vivo *genetic recombination between these two viruses may occur.

Recombination is a frequent event among alphaherpesviruses, enhancing their diversity and modifying virus replication properties *in vivo *[7]. Although the process of interspecific recombination appears inefficient relative to intraspecific recombination [8], a natural interspecies recombinant virus between equid herpesviruses 1 (EHV-1) and 4 (EHV-4) has been recently reported after analyzing EHV-1 field strains [9]. Both the recombinant virus and the parental EHV-4 were found to be non-neuropathogenic in the hamster model [9].

**Figure 1:**
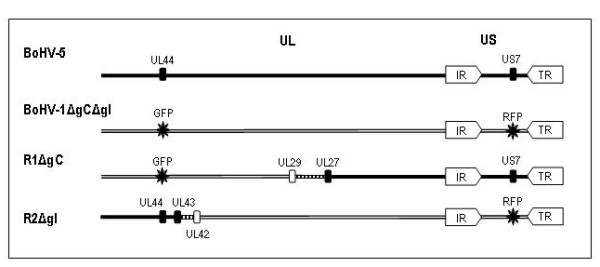
**Representation of the genetic background of the four viruses inoculated in this study**. Parental strains: wild-type BoHV-5 (N569 strain) and mutant BoHV-1ΔgCΔgI; interspecific recombinants: R1ΔgC and R2ΔgI. The organization of the four genomes includes a unique long (UL) and a unique short (US) sequence. The latter is flanked by two repeated and inverted sequences (internal repeat, IR; terminal repeat, TR). The parental BoHV-1 is a mutated strain in which the genes encoding the glycoproteins gC (UL44) and gI (US7) were replaced by sequences encoding fluorescent proteins GFP and RFP respectively (labeled by large asterisks). The BoHV-5 and BoHV-1 sequences are represented by black or white lines and rectangles respectively. The site of recombination in each recombinant virus is represented by discontinuous lines.

Regarding bovine alphaherpesviruses, two recombinant viruses have been isolated from *in vitro *interspecific recombination between a genetically modified strain of BoHV-1 (BoHV-1.2ΔgCΔgI) and BoHV-5 strain N569 [8]. The genetic composition of these recombinants, named R1ΔgC and R2ΔgI, has been published recently [10]. R1ΔgC is composed mainly of BoHV-5 (63%) whereas R2ΔgI is 86% homologous to BoHV-1 (Figure 1). Recombination could have a deep impact on BoHV-1 and BoHV-5 pathogenesis and epidemiology since it could lead to virulence restoration of attenuated strains or to an increase in pathogenicity by acquisition of virulence-associated genes. However, the consequences of recombination on R1ΔgC and R2ΔgI *in vivo *behavior have not been studied yet. The aim of this study was to assess the *in vivo *behavior of two BoHV-1/-5 interspecific recombinant viruses in calves. This study constitutes the first report on the evaluation of interspecific recombinants in the natural host.

## Methods

### Viruses and cell culture

The viruses used in this study are illustrated in Figure 1. Viral stocks -with high number of passages- were kindly provided by Virology and Immunology laboratory of University of Liege. The viruses were propagated in Madin Darby bovine kidney (MDBK, ATCC, USA) cells at a low multiplicity of infection as previously described [11]. Total DNA (viral and cell DNA) was prepared from infected cells using an extraction buffer 2× (Tris 200 mM pH 8, EDTA 20 mM pH 8, NaCl 200 mM, SDS 2%, 2-mercaptoethanol 20 mM) (Sigma-Aldrich, USA) and precipitated by alcohol.

### Animal experiments

Eighteen 3-month-old calves of both genders were randomly separated in four groups of four animals each and one control group of two animals. Their naïve status for BoHV-1 and BoHV-5 exposure was verified by an enzyme-linked immunosorbent assay (ELISA) and seroneutralization assays (SN) [11] and virus isolation from nasal samples upon arrival and before experimental infection. After infection, all groups were strictly isolated from each other in order to prevent spread and contamination with the different virus strains used. Animal care and experimental procedures were performed in accordance with the requirements of the National Institute of Agricultural Technology Ethics Committee (INTA, Argentina).

Calves were infected intranasally (aerosolization) with 3 ml containing a total dose of 10^6.5 ^TCID_50_/ml (1.5 ml in each nostril) of the corresponding viral strain. The control group received 3 ml of cell culture medium. Animals were examined both before the start of the experience and daily after infection by a veterinarian who was not aware of the treatment of individual animals. Respiratory and nervous signs and rectal temperature were recorded. Between days 35-37 post-infection (pi) animals were sedated with acepromazine (Acedan, Holliday Scott S.A., Argentina) by the intramuscular route and then euthanized by barbiturate overdose (Euthanyle, Brouwer, Argentina). Necropsy was performed immediately and encephalon and trigeminal ganglia were dissected.

### Sampling procedure, serological and virological analysis

Blood samples were taken on the day of inoculation and then weekly. Serum antibodies against BoHV-1 were detected by using an indirect ELISA and by SN assays [11].

Nasal samples were collected daily from day 0 to day 18 pi and then twice a week until the end of the experiment and inoculated onto MDBK cell monolayers immediately after collection. Titers were calculated by the Reed and Muench method. Three blind passages were performed on negative samples. Parental and recombinant viral strains were identified either by observation of infected cells with an epifluorescent microscope or by PCR assays using total DNA extracted from infected cells [10]. IgA antibodies in nasal secretions were determined by ELISA as previously described [11].

### Histopathology and detection of latent BoHV DNA in trigeminal ganglia

Samples of CNS (olfactory, frontal, parietal and occipital areas, thalamus, midbrain, pons, obex and cerebellum) and trigeminal ganglion were fixed in 10% buffered formaline solution, embedded in paraffin, sectioned at 4 μm and stained with hematoxylin-eosin (H/E) for histopathological examination.

Pieces of approximately 100 mg of trigeminal ganglion stored at -70°C were digested and total DNA was extracted using the QIAamp DNA Mini kit (Qiagen, Tecnolab S.A., Argentina). Total DNA was subjected to two PCR reactions [12,13] in order to examine the presence of viral DNA. PCR was also performed with a set of primers generating a 250 bp fragment from the bovine glyceraldehyde-3-phosphate dehydrogenase gene (GAPDH), selected as the bovine housekeeping gene. The primers were designated as GAPDH-For (5'-GCA TCG TGG AGG GAC TTA TGA-3') and GAPDH-Rev (5'-GGG CCA TCC ACA GTC TTC TG-3').

## Results

### Clinical and viral response after intranasal infection of cattle

In order to study the *in vivo *behavior of the BoHV-1/-5 recombinant viruses R1ΔgC and R2ΔgI (Figure 1), two groups of four animals were intranasally inoculated as described in Materials and Methods. Two other groups were infected accordingly with the parental strains, BoHV-5 and BoHV-1.2ΔgCΔgI. A mock infected control group of two animals was included.

Calves infected with R1ΔgC, R2ΔgI and parental BoHV-1.2ΔgCΔgI viruses did not show clinical signs or body temperature variations during the period studied (Table 1).

**Table 1:** Viral excretion and humoral response after intranasal infection. Detection of viral DNA in trigeminal ganglia (TG)

	Acute phase			Latent phase	Humoral response	
**Group**	**Nasal excretion**^a^	**Days post infection of excretion onset**	**N° excreting days**	**Viral DNA in TG**^b^	**Mucosal IgA**^c^	**Seroconversion**^d^

**BHV-5 N569**	3/4	2-7	4-10	2/4	3/4	3/4

**BHV-1.2 ΔgCΔgI**	1/4	5*	1	2/3	4/4	0/4

**R1ΔgC**	2/4	2-3	1	2/4	3/4	1/4

**R2ΔgI**	1/4	1*	1	1/4	1/4	0/4

**Mock-infected**	0/2	0	0	0/2	0/2	0/2

* Positive samples after 2-3 blind passages in cell culture.

^a ^Number of excreting animals out of total animals in the group. Identity of excreted virus was confirmed by PCR and fluorescence microscopy.

^b ^Number of trigeminal ganglia (TG) with viral DNA out of total TG available.

^c ^Mucosal IgA raised toward BoHV-1/-5. Number of positive animals out of total animals in the group.

^d ^Serum antibodies against BoHV-1/-5. Number of positive animals out of total animals in the group.

Viral excretion was observed in three out of four animals from the BoHV-5 infected group for 4-10 days, with peak titers of 4.3 to 5.3 log_10 _TCID_50_/ml. In contrast, the tested recombinants and parental BoHV-1.2ΔgCΔgI displayed reduced *in vivo *replication capacities (Table 1). Two calves shed R1ΔgC virus for one day with excretion titers of 2.3 and 2.5 log_10 _TCID_50_/ml, respectively. Concerning the R2ΔgI and BoHV-1.2ΔgCΔgI groups, infectious virus was isolated only after two or three blind passages on cell cultures infected with nasal swabs. The day of excretion onset for each group was variable and is detailed in Table 1. At least one positive isolate was observed in each group.

### Post mortem analysis and establishment of latency

**Figure 2:**
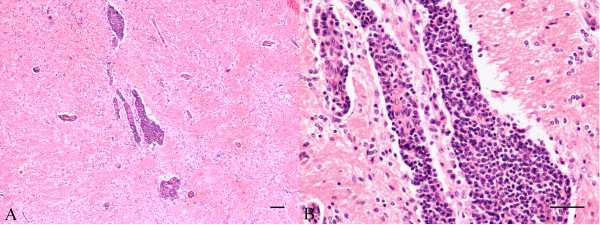
**Histopathological lesions of a BoHV-5 infected calf**. A) Frontal lobe. Mononuclear inflammatory exudate in perivascular cuff. Focal gliosis in white matter. H/E. Bar = 200 μm. B) Frontal lobe. Detail of A: Numerous lymphocytes in perivascular cuff. H/E. Bar = 100 μm.

The histopathological examination performed in CNS samples revealed a severe non-suppurative meningoencephalitis in olfactory, frontal, parietal and occipital cortex, thalamus, midbrain and cerebellum in two of the BoHV-5-infected animals. Lesions were characterized by perivascular infiltration of mononuclear cells (mostly lymphocytes), focal gliosis and focal malacia (Figure 2). In addition, three animals showed slight vascular alterations in the trigeminal ganglion. In contrast, animals infected with R1ΔgC, R2ΔgI and parental BoHV-1.2ΔgCΔgI presented either slight or no histological alterations in all the sections examined.

To investigate the establishment of latency by the different viruses, trigeminal ganglia were dissected after euthanasia 35 days pi. PCR assays were performed to test for the presence of either BoHV-1 or BoHV-5 latent DNA. In addition, trigeminal ganglia were co-cultured with susceptible cells and monitored for BoHV replication. Since no cytopathic effect developed in the cultures, we concluded that the positive PCR results (Table 1) were due to the presence of latent viral genomes. The presence of viral DNA was observed in at least one calf from each of the infected groups, thus indicating that both the recombinant viruses and the parental strains were able to establish a latent infection in trigeminal ganglia.

### Humoral immune response

The immune response specifically raised against BoHV-1 and BoHV-5 after the experimental infection was measured either by ELISA or by the SN assay. Seroconversion was detected in three out of the four BoHV-5-infected animals as well as in one animal from the R1ΔgC group (Table 1). Mucosal IgA was detected in all the infected groups but not in mock-infected calves (Table 1).

In summary, the data show that all the viruses were able to replicate in calves although R1ΔgC, R2ΔgI and parental BoHV-1.2ΔgCΔgI were attenuated *in vivo*. Detectable virus excretion by animals infected with these viruses was low and none of the infected animals developed clinical signs during the experimental infection.

## Discussion

BoHV-1 and BoHV-5 have been shown to co-circulate in nature and cattle co-infection has been recently demonstrated [6]. Even if the process of interspecific recombination appears inefficient relative to intraspecific recombination, a natural interspecies recombinant virus between EHV-1 and EHV-4 has been reported by analyzing EHV-1 field strains [9]. Therefore, BoHV-1 and BoHV-5 may give rise to interspecific recombinants with unknown biological characteristics in co-infected cattle. Recombination may lead to virulence restoration of attenuated strains or to a decrease in the pathogenicity of parental viruses. Regarding this, there are several reports describing the generation of virulent HSV-1 and PrV intraspecific recombinants [14,15] and avirulent HSV-1/-2 interspecific recombinants [16].

The *in vivo *behavior of the *in vitro*-generated interspecific recombinant viruses between BoHV-1 and BoHV-5 was investigated in the natural host. According to our experimental data, after nasal infection of calves, R1ΔgC and R2ΔgI are attenuated but able to replicate at low rates and establish latent infections.

According to the genomic characterization performed elsewhere [10] (Figure 1), most of the genomic background of R1ΔgC virus is homologous to BoHV-5 (63%), including some of the genes associated with the differential pathogenesis between BoHV-5 and BoHV-1 (US7-gI, US8-gE and US9) [2], whereas R2ΔgI virus is 86% homologous to BoHV-1. Despite their genomic content, both recombinant viruses appear to be attenuated in calves. This may suggest that the BoHV-5 genes enclosed in the US region, the internal and terminal repeats and part of the UL region are not able to confer BoHV-5 *in vivo *replication properties to R1ΔgC in the genetic background of the parental BoHV-1. However, since the recombinant viruses were obtained by *in vitro *co-infection between a highly attenuated BoHV-1 strain (tested in this study) and BoHV-5, the evaluation of the association between genetic composition and virulence of these recombinants is limited. Although BoHV-5-infected animals did not present neurological signs, viral excretion and histopathological lesions were observed. Animal age and individual susceptibility, among other factors, could account for the absence of clinical signs in the BoHV-5-infected group [2]. The histological alterations found in the CNS samples from BoHV-5-infected animals were similar to those reported after cattle infection with other BoHV-5 strains [17,18]. The slight lesions observed in both the recombinants and parental BoHV-1 could be due to unspecific cellular alterations.

Serum and/or mucosal antibodies were detected in all groups, consistently with a previous report showing the induction of nasal IgA in calves after infection with different BoHV-1 strains [19]. However, humoral response was not detected in some animals. The low rate of viral replication could account for the lack of induction of immune response. In addition, the sensitivity of the ELISA tests available could be insufficient to detect a very low antibody response evoked by the recombinant viruses.

The reduced virulence observed in the R1ΔgC and R2ΔgI viruses could be attributed to the high attenuation that the parental BoHV-1ΔgCΔgI has *per se*. Indeed, different degrees of virulence have been reported among a variety of BoHV-1 wild type [20] and genetically modified [21] strains. A genetic characterization of the two BoHV-1/-5 recombinants and parental strains has been recently performed in the whole genome [10]. However, other genetic modifications introduced during the generation of the parental double deleted mutant or in the recombinant isolation process could also account for their *in vivo *attenuation. Moreover, small modifications in viral genomes can have a deep impact on the *in vivo *behavior. In this respect, variation of a single amino acid in the polymerase of neuropathogenic EHV-1 strains has been associated with their neurovirulence [22].

The high *in vivo *attenuation that the two BoHV-1/5 recombinants showed in cattle indicates that these recombinants might not constitute a major risk of persistence and spread in cattle population. However, in areas where co-infections with virulent strains of BoHV-1 and BoHV-5 exists (Southern hemisphere) the generation of unknown virulence recombinants could occur.

## Conclusion

In this study we investigated the *in vivo *behavior of two interspecific recombinant viruses generated *in vitro *from closely related alphaherpesviruses infecting the same natural host. Although both recombinants showed reduced virulence following experimental cattle infection, our results demonstrate *in vivo *replication and establishment of latency of *in vitro*-generated BoHV-1/-5 recombinants.

## Competing interests

The authors declare that they have no competing interests.

## Authors' contributions

MPDMZ, SAR, JT, ET and BM designed the experiments, analysed the data and drafted the manuscript. MPDMZ, SAR, MFL and FK performed the experiments. FD carried out the histopathological analysis of the samples. FM and GK helped to draft the manuscript. All authors read and approved the final manuscript.
